# A Case of Adult-Onset Still’s Disease With Hypocomplementemia

**DOI:** 10.7759/cureus.52605

**Published:** 2024-01-20

**Authors:** Yoichi Kurosawa, Sayuri Takamura, Ayako Wakamatsu, Daisuke Kobayashi, Ichiei Narita

**Affiliations:** 1 Division of Clinical Nephrology and Rheumatology, Niigata University Graduate School of Medical and Dental Sciences, Niigata, JPN; 2 Department of Rheumatology, Niigata Rheumatic Center, Shibata, JPN

**Keywords:** hyperferritinemic syndrome, cytokine storm, ferritin, hypocomplementemia, adult-onset still’s disease

## Abstract

Adult-onset Still’s disease (AOSD) causes fever, rash, pharyngalgia, and arthralgia through autoinflammation. Its complement titer has not previously received attention because this usually increases during the inflammatory process. Our female patient in her 60s was admitted to the hospital with fever, rash, arthralgia, and pharyngalgia. Her white blood cell count was 19,130/μL, hemoglobin was 11.0 g/dL, platelet count was 26.0 × 10^4^/μL, and ferritin titer was 6,175 ng/mL. Anti-nuclear antibodies and anti-neutrophil cytoplasmic antibodies were negative. The presence of infectious diseases and malignancies was excluded. She was diagnosed with hypocomplementemia at the onset of AOSD because of her low complement component 4 (C4) titer (<5.0 mg/dL). Her complement component 3 (C3) titer was 104.5 mg/dL, which was within normal limits. There was no sign of thrombotic microangiopathy (TMA) or hemophagocytosis. She was treated with high-dose corticosteroids, including pulse methylprednisolone therapy, cyclosporine, methotrexate, and intravenous immunoglobulin, but was resistant to these, and her disease repeatedly flared up. Treatment with intravenous cyclophosphamide eventually led to remission. Post-treatment, her C4 titer increased to within the normal range. Although hypocomplementemia with TMA or hemophagocytosis has been reported in AOSD patients, our patient showed no sign of either at disease onset. Hypocomplementemia of AOSD may be a sign of high disease activity and could be a predictive marker for resistance to standard therapy.

## Introduction

Adult-onset Still’s disease (AOSD) is a systemic inflammatory disease in which fever; polyarthralgia, which mainly occurs in the wrists, knees, and ankles; pharyngalgia; and evanescent salmon-pink rash, which is mainly shown during fever spikes, are the main clinical symptoms. Levels of C-reactive protein (CRP), neutrophils, liver enzymes, and ferritin are elevated in AOSD patients. The release of several different types of cytokines has been documented in AOSD [1,2], with interleukin (IL)-1β and IL-18 being particularly important. IL-18 was reported to be associated with disease activity and its presence is useful in diagnosing AOSD [3], while IL-1β is important in AOSD etiology and the use of IL-1β inhibitors is a treatment choice for AOSD [4]. The stimulation of these two cytokines [1] leads to the release of many other types in the phenomenon known as a cytokine storm [5-7]. The prevalence rate of AOSD is one to 34 cases per one million people. The peak of AOSD onset is bimodal at ages 15 to 25 and 36 to 46 years [2].

The complement titer of AOSD often rises because of systemic inflammation [8], and there are few reports of AOSD with hypocomplementemia. One of these was a case of AOSD complicated by thrombotic microangiopathy (TMA) [9] in which low complement component 3 (C3) and B factor and normal complement component 4 (C4) were observed, suggesting an alternative pathway activation. Another case report described a patient with acute anemia, thrombocytopenia, hyperferritinemia, and hypocomplementemia [10]. Hemophagocytosis was not seen in the bone marrow, but it was present in the peripheral blood. In these two reports, the etiology of hypocomplementemia was not well studied, and its clinical importance was unknown.

Here, we report an AOSD patient with high disease activity associated with hypocomplementemia but no signs of hemophagocytosis or TMA; the disease was very refractory to treatment. The presence of hypocomplementemia may therefore be a predictive indicator of resistance to standard treatment.

## Case presentation

A woman in her 60s was admitted to our hospital with fever, rash, systemic arthralgia, and pharyngalgia. She had total hip arthroplasty one year previously, and her son had had systemic lupus erythematosus. Two weeks before admission, she developed erythema, which was successfully treated with prednisolone (PSL) 20 mg for four days in the first hospital. Soon after that, she experienced pharyngalgia, systemic arthralgia, and fever with a temperature over 38℃. Anti-microbials were prescribed but were ineffective. Blood analysis showed elevated liver enzymes (aspartate aminotransferase 55 U/L, alanine aminotransferase 75 U/L), CRP (16.53 mg/dL), and ferritin (11,057 ng/mL). Based on these findings, she was suspected of having AOSD and was referred to our hospital.

**Figure 1:**
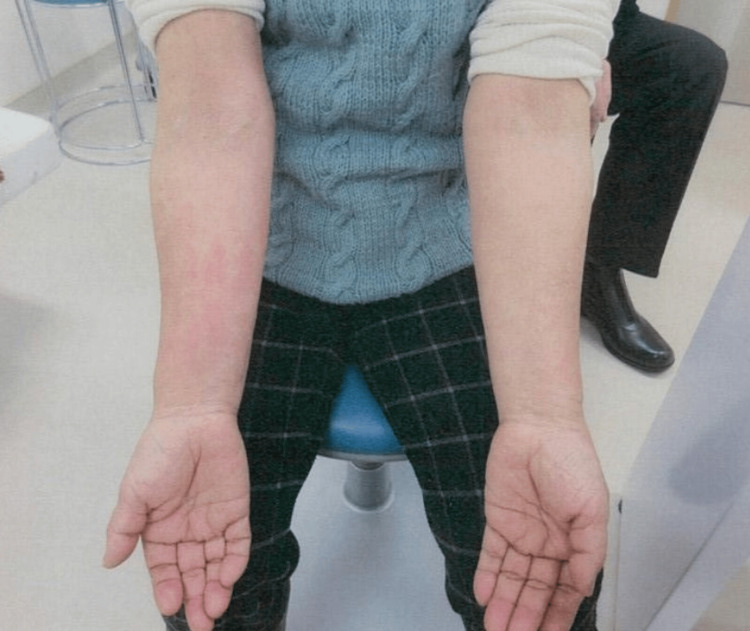
Salmon-colored rash on her right arm on adimisson.

On admission, she had a high fever and salmon-colored rash on her right arm and abdomen (Figure 1). Her joint pain was generalized, including hand and knee joints, but no joint swelling. Her white blood cell count was 19,130/µL, and her neutrophil percentage was 92.9%. Her hemoglobin was 11.0 g/dL, ferritin was 6,175 ng/mL, CRP was 21.71 mg/dL, and platelet count was 26.0 × 10^4^/µL. Levels of aspartate aminotransferase, alanine aminotransferase, and creatinine were 66 U/L, 78 U/L, and 0.52 mg/dL, respectively. Unlike standard AOSD, she showed hypocomplementemia with 50% hemolytic complement activity (CH50) 14 U/mL and C4 <5.0 mg/dL. Her C3 was 104.5 mg/dL, which was within normal limits, and anti-nuclear antibody, rheumatoid factor, and myeloperoxidase and proteinase-3 anti-neutrophil cytoplasmic antibodies were all negative. Her blood culture was also negative. Other laboratory findings are shown in Table 1, and there was no evidence of hemophagocytosis or TMA. We diagnosed her with AOSD because of her sustained fever, erythema, neutrophilia, pharyngalgia, liver dysfunction, and negative anti-nuclear antibodies and rheumatoid factor, based on the Yamaguchi criteria [8].

**Table 1:** Laboratory findings on admission ESR, erythrocyte sedimentation rate; APTT, activated partial thromboplastin time; PT, prothrombin time; HbA1c, hemoglobin A1c; HDL-C, high-density lipoprotein cholesterol; LDL-C, low-density lipoprotein cholesterol; Ig, immunoglobulin; RF, rheumatoid factor; CH50, 50% hemolytic complement activity; C3, complement component 3; C4, complement component 4; ANA, anti-nuclear antibodies; Abs, antibodies; anti-DNA, anti-deoxyribonucleic acid; anti-Sm, anti-smith; anti-RNP, anti-ribonucleoprotein; anti-SS-A, anti-Sjögren’s syndrome A; MPO-ANCA, myeloperoxidase anti-neutrophil cytoplasmic antibodies; PR3-ANCA, proteinase 3 anti-neutrophil cytoplasmic antibodies; sIL-2R, soluble interleukin-2 receptor; IGRA, interferon-gamma release assays

Laboratory findings														
Parameter	At previous hospital	On admission	At discharge	Normal range	Parameter	At previous hospital	On admission	At discharge	Normal range	Parameter	At previous hospital	On admission	At discharge	Normal range
White blood cells (/μL)	17,300	19,130	5,320	3,300-8,600	Total protein (g/dL)	5.5	6.0	6.0	6.6-8.1	IgG (mg/dL)	805	789	774	861-1,747
Neutrophil (%)	89.6	92.9	77.2	38.0-71.0	Albumin (g/dL)	2.8	2.7	3.6	4.1-5.1	IgA (mg/dL)	101	105	21	93-393
Eosinophil (%)	0.3	0.4	0.2	<7.3	Aspartate aminotransferase (U/L)	55	66	19	13-30	IgM (mg/dL)	67	73	32	50-269
Basophil (%)	0.5	0.1	0.0	<2.0	Alanine aminotransferase (U/L)	75	79	11	7-23	RF (IU/mL)	26.2	12.9	N/A	<15.0
Monocyte (%)	1.2	0.9	4.9	3.0-8.0	γ-Glutamyl transpeptidase (U/L)	94	98	26	9-32	CH50 (U/mL)	N/A	14	N/A	30-46
Lymphocyte (%)	8.4	5.7	17.7	21.0-55.0	Alkaline phosphatase (U/L)	315	366	89	106-322	C3 (mg/dL)	108.3	104.5	71.8	73.0-138.0
Red blood cells (×10^4^/μL)	354	377	367	386-492	Lactate dehydrogenase (U/L)	370	423	231	124-222	C4 (mg/dL)	8.7	<5.0	16.5	11.0-31.0
Hemoglobin (g/dL)	11.0	11.0	12.0	11.6-14.8	Total bilirubin (mg/dL)	0.53	0.7	0.8	0.4-1.5	ANA (Index)	N/A	6.9	N/A	<20
Platelet (×10^4^/μL)	32.6	26.0	21.4	15.8-34.8	Creatine kinase (U/L)	21	15	39	41-153	Anti-DNA Abs (IU/mL)	N/A	1	N/A	<12
ESR (nn/hr)	N/A	57	N/A	3-15	Triglyceride (mg/dL)	110	97	165	30-117	Anti-Sm Abs (C.O.I)	N/A	<5.0	N/A	<7.0
APTT (sec.)	26.9	25.9	N/A	26.9-40.9	HDL-C (mg/dL)	31	32	70	48-103	Anti-RNP Abs (C.O.I)	N/A	<5.0	N/A	<15.0
PT% (%)	98.2	94	N/A	70-130	LDL-C (mg/dL)	89	96	184	65-163	Anti-SS-A Abs (C.O.I)	N/A	<5.0	N/A	<10.0
Fibrinogen (mg/dL)	392.4	468	N/A	200-400	Ferritin (ng/mL)	11107	6175	23	5-152	Anti-Jo-1 Abs (C.O.I)	N/A	<5.0	N/A	<9.0
D-dimer (μg/mL)	8.1	4.6	N/A	<1.0	C-reactive protein (mg/dL)	16.53	21.71	<0.01	<0.14	MPO-ANCA (U/mL)	N/A	<1.0	N/A	<3.5
Glucose (mg/dL)	124	173	91	70-140	Blood urea nitrogen (mg/dL)	13.7	11	16	8-20	PR3-ANCA (IU/mL)	N/A	<0.5	N/A	<3.5
HbA_1_c (%)	6.0	6.0	5.1	4.9-6.0	Creatinine (mg/dL)	0.55	0.52	0.62	0.46-0.79	sIL-2R (U/mL)	N/A	2096	N/A	122-496
					Sodium (mmol/L)	133	132	142	138-145	IGRA	N/A	(-)	N/A	(-)
					Potassium (mmol/L)	4.06	4.2	3.5	3.6-4.8					
					Chlorine (mmol/L)	99	99	106	101-108					

**Figure 2:**
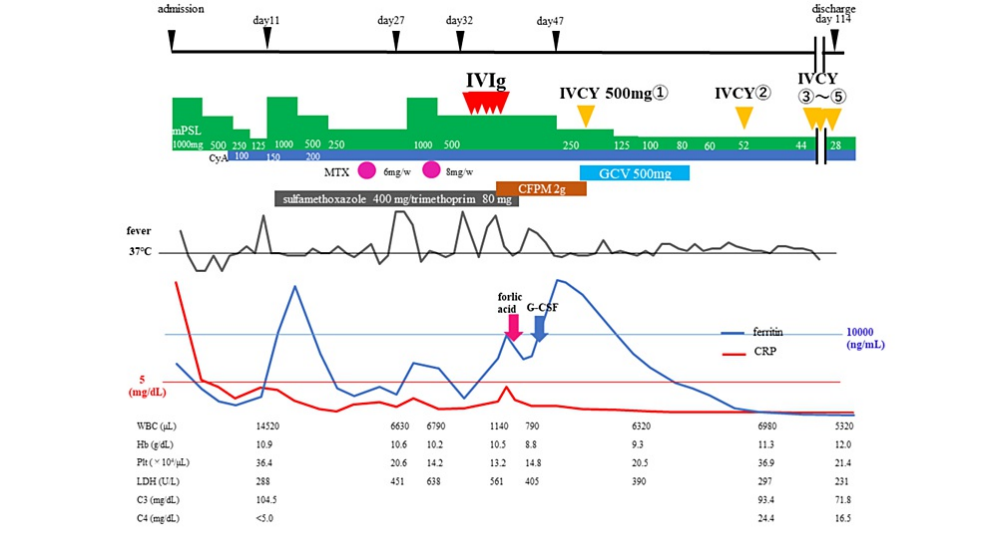
Clinical course of the patient after admission The patient was treated with several rounds of corticosteroid pulse therapy and various immunosuppressive drugs. Low C4 levels were seen at the onset of disease increased after the treatment. mPSL, methylprednisolone; IVIg, intravenous immunoglobulin; IVCY, intravenous cyclophosphamide; CyA, cyclosporine; MTX, methotrexate; CFPM, cefepime; GCV, ganciclovir; G-CSF, Granulocyte colony stimulating factor; CRP, C-reactive protein; WBC, white blood cells; Hb, hemoglobin; Plt, platelet; LDH, lactate dehydrogenase; C3, complement component 3; C4, complement component 4

Her clinical course during hospitalization is shown in Figure 2. After diagnosis, treatment with pulse methylprednisolone (mPSL) therapy of 1 g/day for three days was started. The mPSL dose was then tapered, and cyclosporin A (CsA) 100 mg/day was added. However, on day 11, when the dose of mPSL was reduced to 125 mg/day, fever, pharyngalgia, and fatigue returned, with elevated titers of ferritin and CRP, leading to a diagnosis of AOSD relapse. We increased the dose of mPSL up to 1 g/day before tapering it again and adding methotrexate (MTX) 6 mg/week. Despite these large quantities of mPSL and two immunosuppressants, fever recurred again, and the CRP titer was elevated on day 27. We administered pulse mPSL therapy of 1 g/day for the third time and added intravenous immunoglobulin therapy (IVIg) 20 g/day because of her resistance to standard treatment. However, fever again returned, and neutropenia and hyperferritinemia were observed. We suspected hemophagocytosis and MTX-induced bone marrow suppression, so we administered leucovorin 24 mg/day, plus filgrastim 75 μg/day and cefepime 2 g/day for febrile neutropenia. The addition of IVIg was also ineffective, so intravenous cyclophosphamide (IVCY) 500mg was started. No relapse occurred, even after the mPSL dose was reduced, and IVCY was administered a total of five times every two to three weeks. The dose of mPSL was reduced to 28 mg/day and the patient was discharged. On day 59, C3 was 93.4 mg/dL and C4 was 24.4 mg/dL. After discharge, she was followed up for three years. During this time, she was treated with CsA 250 mg/day and mPSL, the latter being decreased to 4 mg/day. Her C3 was around 80 mg/dL, and C4 was around 15 mg/dL. She has been doing well with no major sequelae or complications, and no AOSD relapse has been observed.

## Discussion

We report a patient with AOSD who presented with hypocomplementemia at the onset of disease, despite AOSD being an inflammatory disease in which the complement is usually elevated. The present case also had high disease activity and was resistant to standard therapy.

In patients with AOSD, systemic inflammation usually causes the complement titer to rise together with CRP levels and the erythrocyte sedimentation rate. Because elevated complement levels do not have a specific clinical significance, complement titers are typically not the focus of attention in the clinical management of AOSD. However, some AOSD cases have presented with hypocomplementemia, although they were further complicated with hemophagocytosis [10] or TMA [9,11,12]. These cases are shown in Table 2. These are rare cases in AOSD patients. Common causes of hypocomplementemia include systemic lupus erythematosus, Sjogren's syndrome, cryoglobulinemia, membranoproliferative glomerulonephritis, post-streptococcal glomerulonephritis, rheumatoid vasculitis, liver failure, and complement deficiency, and AOSD is not a common cause. In a previously reported case of AOSD with hemophagocytosis and hypocomplementemia [10], Gorelik et al. presumed that hypocomplementemia resulted from a urokinase-like plasminogen activator produced by activated macrophages. This case was also referred to in a review of cytokine storms, where hypocomplementemia was attributed to the consumption of immune complexes [6]; however, the involvement of immune complex consumption in AOSD has not been documented in the previous research.

**Table 2:** Previously published cases of adult-onset Still’s disease showing hypocomplementemia WBC, white blood cells; Hb, hemoglobin; Plt, platelet; C3, complement component 3; C4, complement component 4; TMA, thrombotic microangiopathy; CS, corticosteroid; PEX, plasma exchange; HD, hemodialysis Citations: case 1 [10], case 2 [9], case 3 [11], case 4 [12]

	Age	Sex	Complication	WBC	Hb	Plt	Ferritin	C3	Normal range of C3	C4	Normal range of C4	Treatment
Case 1	17	Female	Hemophagocytosis	13,500 μL	8 g/dL	4.3×10^4 ^μL	12,558 ng/mL	20 mg/dL	79-152 mg/dL	<5 IU/L	16-38 IU/L	CS
Case 2	46	Female	TMA	10,900 μL	7.7 g/dL	9.8×10^4 ^μL	7420 μg/L	72.4 mg/dL	82.5-114 mg/dL	19.7 mg/dL	15.7-25.7 mg/dL	CS, PEX
Case 3	69	Female	TMA	23,510 μL	7.5 g/dL	7.1×10^4 ^μL	No data	71 mg/dL	85-193 mg/dL	normal	15.7-25.7 mg/dL	CS, PEX, HD, eculizumab
Case 4	26	Female	TMA	6,360 μL	6.4 g/dL	6.3×10^4 ^μL	816 nmol/L	58 mg/dL	79-152 mg/dL	11 mg/dL	16-38 mg/dL	CS, PEX, HD

In patients with AOSD and TMA, decreased C3 levels but normal C4 levels were previously observed [9,11]. Our patient had a notable C4 reduction, suggesting that the mechanism of hypocomplementemia differed from that in patients with TMA. To our knowledge, this is the first case of AOSD with hypocomplementemia without obvious hemophagocytosis or TMA.

The etiology of hypocomplementemia was unknown in our patient. One possible mechanism is the decreased production of complement following liver dysfunction. Liver enzyme levels are often elevated in AOSD patients [2], but to our knowledge, there have been no reports of decreased complement production to date. Some diseases that exhibit similar pathological conditions to AOSD, causing cytokine storms and hyperferritinemia, are sometimes referred to as "hyperferritinemic syndromes" [13]. These syndromes include macrophage activation syndrome, Still’s disease, septic shock, catastrophic antiphospholipid syndrome, and COVID-19 [7,13]. Of the hyperferritinemic syndromes, COVID-19 [14] and septic shock [15] were previously reported to show hypocomplementemia, which is related to disease severity or mortality. As seen in severe COVID-19, hypocomplementemia in AOSD might also be associated with cytokine storms and high disease activity. This indicates that the complement value should be determined in AOSD patients, and if hypocomplementemia is observed, they should be treated on the assumption that the disease might be resistant to standard therapy.

AOSD with hypocomplementemia may have high disease activity and be intractable to standard therapy. These standard therapies include corticosteroids, such as pulse glucocorticoid therapy, disease-modifying anti-rheumatic drugs including MTX, tacrolimus, cyclosporine A, and azathioprine, as well as biologics, such as the IL-1 inhibitors canakinumab and anakinra and tocilizumab [16-18]; however, canakinumab and anakinra are not approved for health insurance treatment in Japan. In our case, IVCY [19] and IVIg [20] were needed in addition to a corticosteroid, calcineurin inhibitor, and MTX. These therapeutic drugs have previously been used for patients with AOSD who are refractory to treatment [19,20].

## Conclusions

We report a case with hypocomplementemia at the onset of AOSD. This patient was resistant to standard therapy and her disease repeatedly flared up. The complement titer of AOSD usually increases during the inflammatory process. However, AOSD can present with hypocomplementemia, which could be a marker for high disease activity and resistance to standard therapy. We should carefully observe the complement titer in the AOSD patients before treatment.
